# Neuroinflammation and Scarring After Spinal Cord Injury: Therapeutic Roles of MSCs on Inflammation and Glial Scar

**DOI:** 10.3389/fimmu.2021.751021

**Published:** 2021-12-02

**Authors:** Qi-Ming Pang, Si-Yu Chen, Qi-Jing Xu, Sheng-Ping Fu, Yi-Chun Yang, Wang-Hui Zou, Meng Zhang, Juan Liu, Wei-Hong Wan, Jia-Chen Peng, Tao Zhang

**Affiliations:** ^1^ Key Laboratory of Cell Engineering of Guizhou Province and Regenerative Medicine Centre, Affiliated Hospital of Zunyi Medical University, Zunyi, China; ^2^ Department of Orthopedics, Affiliated Hospital of Zunyi Medical University, Zunyi, China; ^3^ Department of Human Anatomy, Zunyi Medical University, Zunyi, China

**Keywords:** spinal cord injury, mesenchymal stem cells, astrocyte, T cells, macrophage, neuroinflammation, glial scar

## Abstract

Transected axons are unable to regenerate after spinal cord injury (SCI). Glial scar is thought to be responsible for this failure. Regulating the formation of glial scar post-SCI may contribute to axonal regrow. Over the past few decades, studies have found that the interaction between immune cells at the damaged site results in a robust and persistent inflammatory response. Current therapy strategies focus primarily on the inhibition of subacute and chronic neuroinflammation after the acute inflammatory response was executed. Growing evidences have documented that mesenchymal stem cells (MSCs) engraftment can be served as a promising cell therapy for SCI. Numerous studies have shown that MSCs transplantation can inhibit the excessive glial scar formation as well as inflammatory response, thereby facilitating the anatomical and functional recovery. Here, we will review the effects of inflammatory response and glial scar formation in spinal cord injury and repair. The role of MSCs in regulating neuroinflammation and glial scar formation after SCI will be reviewed as well.

## Introduction

**Figure 1:**
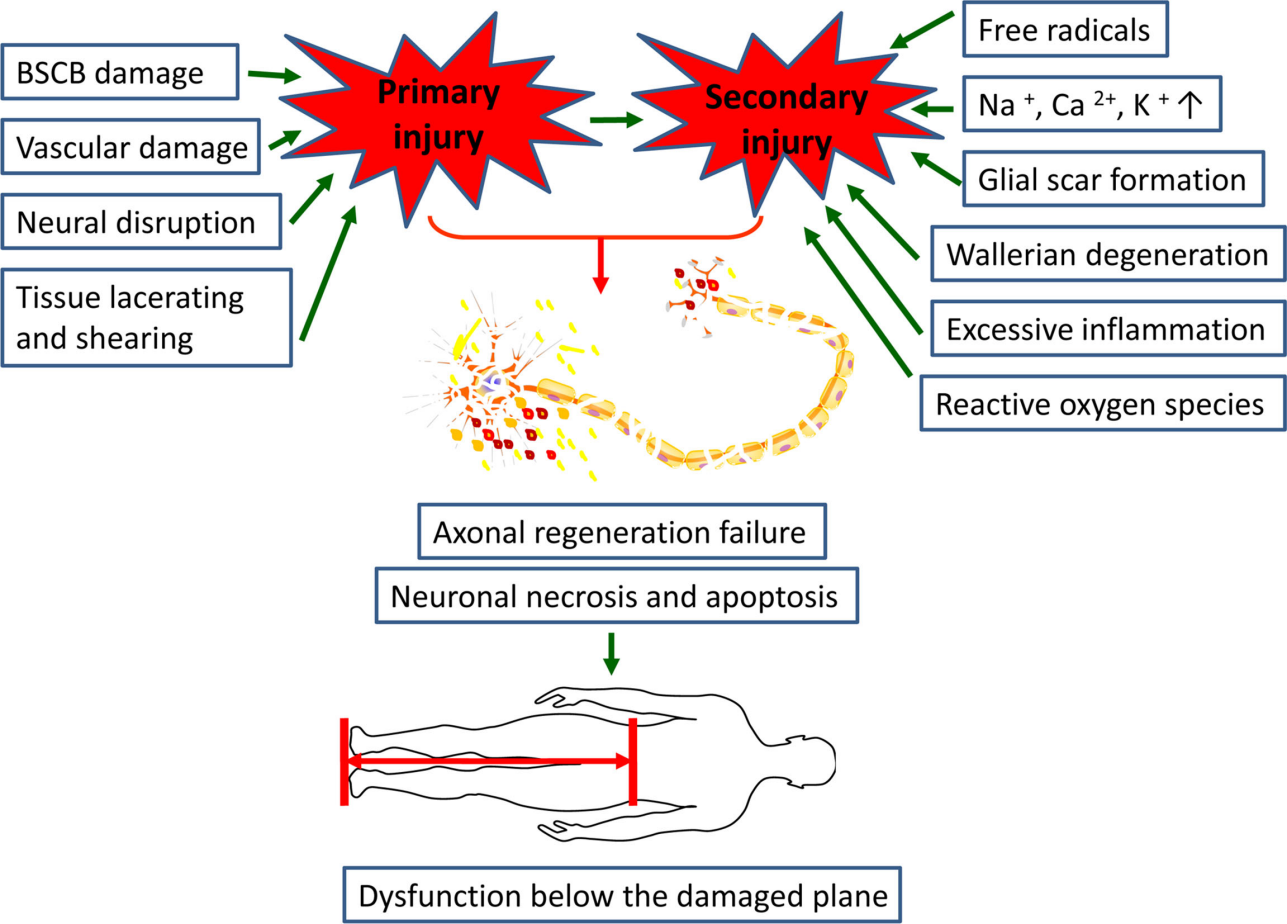
Primary and secondary injuries together leading to axonal regeneration failure and neuronal necrosis and apoptosis, results in dysfunction below the damaged plane in SCI patients.

Spinal cord injury (SCI) is a permanent and disabling disorder that generates great personal loss and social burden (1). It is estimated that the global incidence of SCI ranges from 10.4 and 83 per million per year, and will continue to rise with the rapid development of transportation and aging population (2, 3). In general, we customarily divide the pathophysiology process of SCI into primary injury and secondary injury; the former commonly occurs owing to vertebral fracture or dislocation caused by a mechanical insult, which would destroy the nervous tissue directly (4, 5). Following the primary injury, ongoing pathological changes contribute to secondary injury, which is mainly characterized by chronic inflammation, cell dysfunction, vascular changes, etc. (6). Primary and secondary injury events not only activate resident astrocytes and microglia, fibroblasts, and other glial cells, but also contribute to the infiltration of peripheral immune cells, and the interaction between these cells underlies the glial scar, inflammation, ionic imbalance, and free radical formation, which together inhibit the formation of axonal regeneration and myelination (**Figure 1**) (7, 8).

Both initial traumatic injury and secondary events cause damage of the neuronal conduction pathways, and then result in the deficits of a range of senses and movements below the damaged plane (9). The deficiency in functions can not only severely reduce the quality of life of patients suffering from SCI, but also has a devastating influence on their families and society because it can keep them from engaging in economic activities (10). Therefore, therapies targeting SCI are to rebuild the integrity of damaged neurons, as well as avoid the secondary damage to more healthy nerve tissue surrounding the lesion (11). However, numerous studies have documented that inflammatory cells will persist in the lesion area post-SCI, and these cells further aggravate the injury of the surrounding normal spinal cord tissue by secreting various inflammatory cytokines, reactive oxygen species and proteolytic enzymes, leading to more serious neurological dysfunction (12, 13). In addition, glial scar in the later stage forms a physical and biochemical barrier to axon regeneration (14). Therefore, many scholars believe that the ideal treatment method for SCI is to reduce the glial scar levels in the later stage, thus creating a permissive environment for axon regrow (15).

Over the past decade, an increasing body of evidences has documented that mesenchymal stem cells (MSCs) engraftment can be served as an effective therapeutic strategy for SCI (16, 17). Early on, it was expected that these multipotent cells were likely to promote functional recovery *via* their multidirectional differentiation potential, which may enable them to replace injured neural tissue (18). However, to date, the differentiation of MSCs into neurons *in vivo* still remains controversial (19). Notably, MSCs transplantation could still promote functional and anatomical recovery in the SCI models, and subsequent studies found that this phenomenon might mainly depend on their powerful paracrine effect and direct cell-to-cell contact, which involved in the regulation of immune cells and inflammatory cytokines (20, 21). Furthermore, dozens of evidences indicated that transplanted MSCs could modulate the formation of glial scar *via* changing the level of cytokines, thus promoting axonal regeneration, inhibiting cavity formation, and finally promoting functional recovery (22). Here, we will review the effects of inflammatory response and glial scar formation on SCI prognosis. Meanwhile, we will pay special attention to the therapeutic role of MSCs on neuroinflammation and glial scar formation.

## Inflammatory Response After SCI

Inflammatory response is an integral component of secondary responses because it directly or indirectly determines the outcome of SCI (23). A mass of studies has revealed that early inflammatory response is beneficial, as it removes tissue debris and elevates the level of neurotrophic factors (13, 24). However, when the inflammatory response persists, inflammatory cells will release a great amount of inflammatory cytokines, proteolytic enzymes, matrix metalloproteinases, reactive oxygen species (ROS), leading to more damage to the surrounding healthy spinal cord tissue (25–28). The blood-spinal cord barrier (BSCB) is mainly composed of nonporous capillary endothelial cells, basal cells, pericytes and astrocytes, which together not only prevent the central nervous system (CNS) from the metabolic wastes and neurotoxic molecules in the circulating blood, but also regulate the nutritional molecules into the brain (29, 30). These functions play a significant role in maintaining the homeostasis of CNS and normal operation of the neural function (31). However, one of the earliest events following SCI is the disruption of the integrity of the BSCB and damage of the neural cells (29). Damaged-nerve cells produce large amounts of cellular debris and intracellular proteins, called damage-associated molecular patterns (DAMPs), which bind to pattern recognition receptors (PRRs) on the surface of resident astrocytes and microglia, causing these inflammatory cells to release large amounts of cytokines, chemokines, and reactive oxygen species (32, 33).

After the destruction of the BSCB, peripheral immune cells are driven to the lesion site by the various chemokines and cytokines (34, 35).These infiltrating peripheral immune cells will release some similar soluble factors, making them be the primary cellular source of these molecules at the lesion site (36). The first peripheral immune cells to infiltrate the injury site are neutrophils, which reach their peak at about 24h post-SCI (30). And study has revealed that the number of infiltrating neutrophils was correlated with the severity of SCI (37). These cells can not only facilitate the phagocytosis and removal of cell debris, but also promote repair by secreting leukocyte protease inhibitors (38). However, these cells can also release large amounts of pro-inflammatory cytokines, proteolytic enzymes, and reactive oxygen species, which enhance the inflammatory response and lead to myelin degradation, aggravating the necrosis and apoptosis of damaged neurons (39). It has been shown that activation and infiltration of IKK-β-dependent neutrophils in the injured spinal cord exacerbates neuroinflammation and neuronal damage, thereby impeding functional recovery after SCI (40). More researches are needed to elucidate the role of neutrophils in spinal cord injury and repair.

After neutrophils infiltration, macrophages are the next immune cells to present in the lesion site, and peak within the spinal cord approximately 7 days post injury (41). This cell type was derived initially from resident microglia and subsequently mainly from circulating monocytes. However, these two types of macrophages from different sources are very similar in morphology, gene expression, as well as relative function, so it is difficult to distinguish them at the lesion site (42, 43). As a major component of innate immune response, macrophages have been regarded as an important cell type in regeneration context post-SCI due to their strong functional plasticity (44). Studies have revealed that these immune cells can not only maintain homeostasis by regulating tissue repair and metabolic activities, but also play a central role in immune response and host defense (45). Based on the differences of cell surface markers, gene expression and secretory soluble factors, macrophages are categorized into two main subtypes named M1 and M2: pro-inflammatory cytokines, like interleukin-6 (IL-6) and tumor necrosis factor alpha (TNF-α), ROS, proteolytic enzyme by M1 and anti-inflammatory cytokines, like IL-10 and TGF-β1 by M2 (46, 47). M1 macrophages act as the bad guys by promoting inflammatory response, inhibiting axons regeneration and demyelination. In contrast, anti-inflammatory M2 macrophages play a protective role in promoting functional recovery by removing apoptotic cells and promoting axonal regeneration and myelin sheaths (48, 49). During the process of skin and muscle wounds healing, macrophages will shift from M1 to M2 phenotype in response to the change of microenvironmental stimulus signals at the lesion site (50, 51). Whereas, an analogous M1 to M2 conversion was not observed in the process of spinal cord tissue repair, and the persistent presence of M1 macrophages would exacerbate secondary injury (52–54). Therefore, inducing macrophages switch towards M2 phenotype following acute inflammatory response may contribute to anatomical and functional recovery post-SCI.

Astrocytes, although not immune cells, have been shown to play a crucial role in the innate and adaptive immune responses post CNS injury (55). Study has documented that locally increased IL-1β could mediate the synthesis of MCP-1, KC, and MIP-2 *via* activating the MyD88/IL-1R1 signaling in astrocytes, and these chemokines could result in the infiltration of neutrophils and monocytes, thereby triggering neuroinflammation at the lesion site (56). Moreover, following CNS injury, activated astrocytes would express and secret a wide range of molecules, including chemokines, inflammatory cytokines, adhesion molecules, and nitric oxide, which together create a pro-inflammatory microenvironment. However, astrocytes inhibited in NF-κB signaling significantly reduced the expression of pro-inflammatory and oxidative stress genes, thus exerting a neuroprotective effect (57). Zamanian et al. (58) found that astrocytes could differentiate into two subtypes, termed A1 and A2 astrocytes, under two different conditions of neuroinflammation and ischemia. A1 phenotype greatly upregulates the expression of many gene, like classic complement cascade genes, which have been shown to have destructive effects on synaptic formation, whereas A2 phenotype contributes to axonal regeneration and neuroprotection by upregulating the expression of neurotrophic factors and anti-inflammatory cytokines (59–61). Glial fibrillary acidic protein (GFAP), a cytoskeleton protein found in astrocytes, has been used as a specific marker for astrocytes (62). Complement component 3 (C3) is a bio-marker for A1 astrocytes but not expressed by A2 astrocytes, so C3 + GFAP has been used to identify A1 phenotypes. A2 astrocytes have been found to specifically express S100A10, a member of the S100 protein family, so S100A10 + GFAP was used as a double-marker for detecting A2 phenotype (63, 64).

T lymphocytes are activated ensuing SCI and play an important role in the neuroinflammation and downstream cascades of nerve degeneration and repair (65). Serpe et al. (66) found that the survival of facial motor neurons after axonectomy depended on the presence of anti-inflammatory CD4+ T cells, and their analysis of postoperative T cell subsets revealed that both pro-inflammatory cells (Th1 and Th17) and anti-inflammatory cells (Th2 and Tregs) were activated after injury. The balance between T cell subtypes is essential for nerve tissue repair. However, during SCI, the balance between Th1/Th2 and Th17/Tregs is disrupted, and the adaptive immune response is biased towards the pro-inflammatory Th1 and Th17 phenotypes, leading to increased release of numerous inflammatory cytokines such as interferon gamma (IFN-γ), TNF-β, and IL-17 (4, 29, 67). In addition, these two cell types promoted B lymphocytes to synthesize and release autoantibodies, which further contributed to neuronal demyelination and axonal damage (4, 65). The deficiency of miR-155 significantly inhibited the differentiation of CD4+ T cells to Th17 cells after SCI and reduced the expression of the pro-inflammatory cytokine (IL-17), thus promoting the functional recovery post SCI (68). Therefore, inducing a shift of T lymphocyte towards Th2 and Tregs phenotype may be beneficial to play a neuroprotective role in the early stage of SCI.

In summary, numerous researches have been performed on the activation, proliferation, and polarization of resident and peripheral immune cells for their great contribution to the neuroinflammation post-SCI. Early inflammatory events are essential for clearing cellular debris and pathogens and limit the extent of acute injury. However, following this acute phase, the excessive inflammatory response can hinder the axonal regeneration, new neuronal growth and myelin sheath germination, resulting in severe neurological dysfunction. Therefore, inhibiting these immunological responses of the damaged spinal cord has become the major therapeutic strategies to protect against apoptosis and oxidative damage, and promote angiogenesis as well as neuronal regeneration.

## Role of MSCs on Inflammatory Response

**Figure 2:**
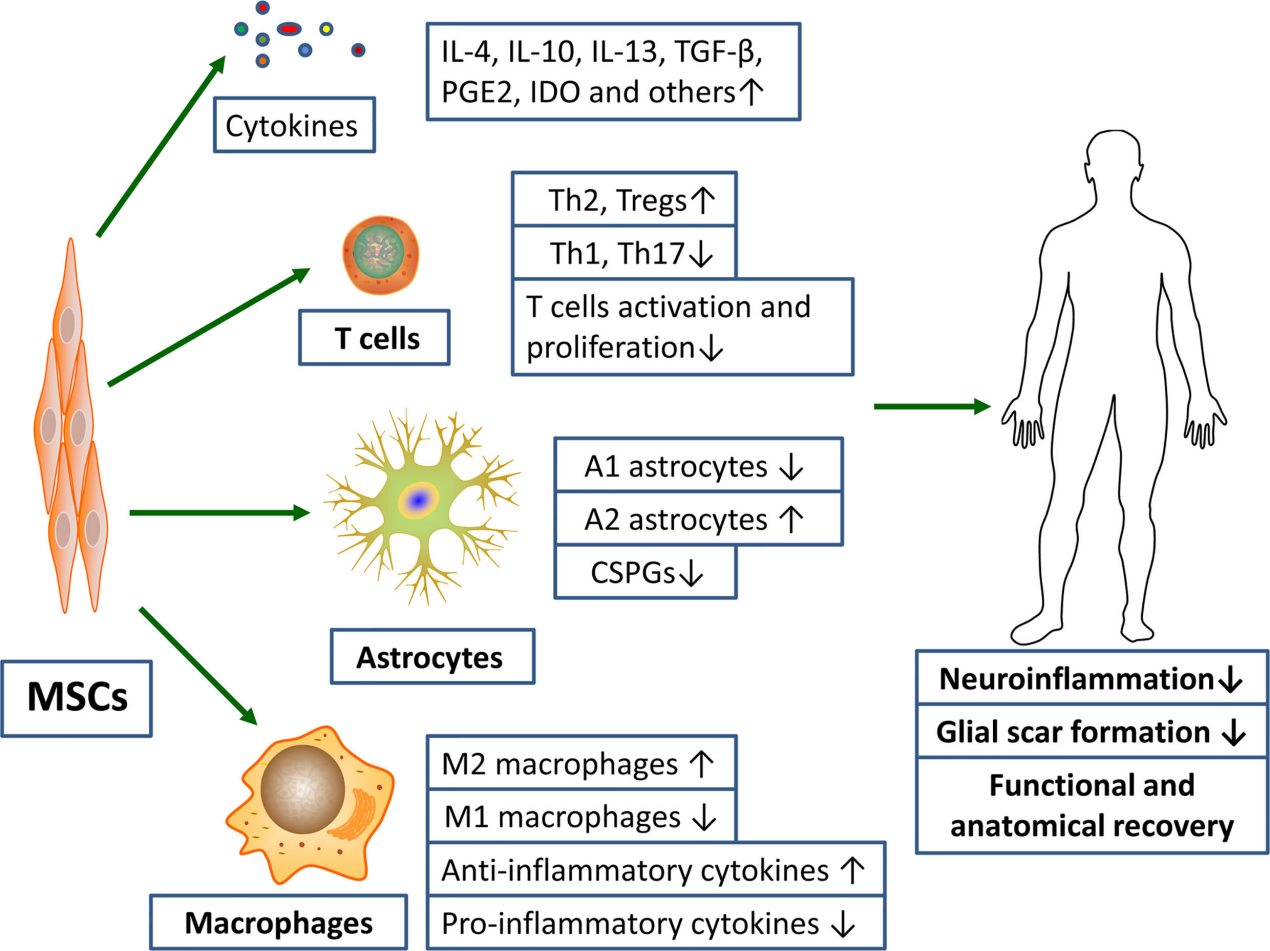
MSCs transplantation promotes functional and anatomical recovery in SCI patients by inhibiting excessive inflammatory response and glial scar formation. Notes: ↑, promotion; ↓, inhibition.

Currently, therapies targeting post-SCI neuroinflammation are extremely limited. The only drug approved by FDA and used in clinical treatment is methylprednisolone sodium succinate, whose main mechanism of action is binding to glucocorticoid receptors to prevent nuclear translocation of pro-inflammatory transcription factors (26, 69). Whereas, the clinical application of hormone has declined over the past few decades due to its unclear therapeutic value and its associated serious complications, such as gastrointestinal bleeding, aseptic necrosis of the femoral head, and wound infection (70, 71). With the rapid development of regenerative medicine, scientists have isolated various MSCs from different tissues, such as peripheral blood, bone marrow, placenta, umbilical cord and amniotic fluid (72–74). And numerous studies have showed that these pluripotent stem cells can effectively correct various functional parameters of the SCI models (**Figure 2**) (9). In investigating the underlying mechanisms involved, it was found that MSCs exert their therapeutic effects mainly through the cell-cell interactions and the secretion of various cytokines to suppress the inflammatory response (75).

Studies have shown that transplantation of bone marrow mesenchymal stem cells (BM-MSCs) into SCI rat contusion models could significantly up-regulate the number of M2 macrophages and down-regulate the number of M1 macrophages at the injury site, accompanied by increased levels of IL-4 and IL-13, and decreased levels of TNF-a and IL-6, which might contribute to the recovery of motor function, increased retention of axon and myelin sheath as well as less glial scar formation after injury (76). Considering the adverse effects of toxic microenvironment on the survival rate and efficacy of MSCs following SCI, biomaterials that can provide neurotrophic factors, protective growth factors, drugs or nanoparticles are co-transplanted with MSCs to enhance their inhibition of glial scar and promote neuroprotective and anti-inflammatory effects (77, 78). Peripheral blood mesenchymal stem cells (PB-MSCs) have attracted our attention due to their unique advantages of the ease of harvesting samples, along with lesser pain to patients (72). Recently, we transplanted PB-MSCs into the rat SCI contusion models and found that the function of posterior limb locomotion was significantly improved in the PB-MSCs transplantation group, which might correlate with a significant increase in the ratio of M2/M1 macrophages and higher levels of the anti-inflammatory cytokines (IL-10 and TGF-β1), as well as decreased levels of the pro-inflammatory cytokines (IL-6 and TNF-α). Furthermore, we also found similar molecular expression patterns and macrophages polarization while macrophages co-cultured with PB-MSCs *in vitro* (18). Notably, the levels of IL-10 in the co-culture system were significantly increased (18, 79). MSCs are known to produce IL-10 (80, 81). Moreover, studies have shown that IL-10 could mediate the polarization of M2 macrophages by activating JAK/STAT3 (82, 83). Therefore, we speculate that IL-10 secreted by PB-MSCs may mediate the formation of M2 phenotype by activating the JAK/STAT3 signaling in macrophages. However, more studies are needed to for further confirmation.

Exosomes (exo) play a critical role in the immune regulation of MSCs (84). Kaminski et al. (85) found that in brain injury models, MSCs-derived extracellular vesicles significantly upregulated the ratio of A2/A1 reactive astrocytes, accompanied by increased expression of nerve growth factors (i.e., BDNF, VEGF and EGF) and anti-inflammatory cytokines, which together improved the survival of neurons and increased the vascular density. BM-MSCs-exo transplantation into SCI animals showed that they significantly promoted angiogenesis, reduced neuronal apoptosis, inhibited glial scar formation, reduced lesion area, inhibited inflammation and promoted axon regeneration, and also significantly inhibited the polarization of A1 astrocytes (86). In addition, research has revealed that intravenous MSCs and MSCS-exo might reduce the number of A1 astrocytes induced after SCI by inhibiting the nuclear translocation of NF-κBp65, thus exerting the anti-inflammatory and neuroprotective effects (87). We usually regard the amount of IL-6 as an indicator of the degree of inflammation post-SCI because IL-6 can aggravate the secondary inflammation to some extent (88, 89). Whereas, some studies have documented that IL-6 can also weaken the inflammation and promote tissue repair after injury, which may be explained by the fact that it plays a defense mechanism in the acute inflammatory response but in chronic inflammation it mainly presents pro-inflammatory characteristics (90). It was found that MSCs might activate the JAK/STAT3 pathway in astrocytes by secreting IL-6, and then mediate the polarization of neuroprotective A2 astrocytes, thereby reducing the neuron damage post brain injury (91). At present, there are still few studies on the polarization of astrocytes mediated by MSCs. Hence, more experiments are needed to confirm different MSCs effects on the activation of astrocytes and its related molecular mechanisms.

The interaction between MSCs and T cells has been well studied. MSCs could strongly inhibit T cell activation and proliferation both *in vitro* and *in vivo*, and induced distinct cell types with specific phenotypes (92). Researchers have documented that MSCs could inhibit the division of T cells by down-regulating the expression of cyclin D2 and promoting the expression of p27Kip1 as well, leading to cell cycle arrested at the G1 phase (93). Moreover, MSCs could still inhibit the proliferation of T cells when the cells were separated by a Transwell system, suggesting that soluble factors were involved in this phenomenon (94). Studies showed that the inhibitory effect of MSCs on T cell proliferation was mediated by their secretion of transforming growth factor-β1 (TGF-β1), hepatocyte growth factor (92) and prostaglandin E2 (PGE2) (95, 96). Furthermore, indoleamine 2, 3dioxygenase (IDO) and nitric oxide (NO) also played an important role in this process (97, 98). For example, MSCs stimulated by IFN-γ could promote the conversion of tryptophan to downstream metabolite kynurenine by secreting IDO, thereby inhibiting the proliferation of T cell (99). Furthermore, it also found that NO secreted by MSCs could inhibit T cell proliferation by inhibiting STAT5 phosphorylation in T cells (100). Expect for inhibiting T cell proliferation, MSCs could also suppress the proliferative response of Th1 and Th17 cells but induce the formation of Th2 and Treg cells to exert their immunosuppressive activity (101). Studies have shown that BM-MSCs-derived exo could not only induce a shift of Th1 towards Th2 cells, but also down-regulated the ratio of Th17/Treg, accompanied by decreased levels of TNF-α and IL-1β and increased levels of TGF-β (102). Previously, we found that the ratio of CD4 + IL-17 + Th17/CD25 + Foxp3 + Treg decreased when the lymphocytes were directly co-cultured with PB-MSCs (18). Similarly, transplanting PB-MSCs into SCI rats also found that they inhibited the expression of Th17 related genes and facilitated the expression of Treg related genes, which might contribute to the functional recovery (103). In investigating the mechanisms involved, it was found that MSCs inhibited Th17 differentiation from naive and memory T-cell precursors by PGE2 *via* EP4 (104). In addition, studies have also found that ICOSL on MSCs binding to ICOS on CD4+T cells could facilitate the differentiation of Treg cells through the activation of PI3K-Akt signaling (105).

In summary, MSCs exhibit encouraging anti-inflammatory roles on the cellular micro-environment. A large number of animal experiments have shown that they can induce the formation of anti-inflammatory immune cells such as M2 macrophages, A2 astrocytes, Tregs and Th2 cells, and then inhibit the inflammatory response at the site of injury before maintaining nervous regeneration and reducing neuronal apoptosis. Neuroinflammation after SCI is indeed a double-edged sword and should be treated to promote early beneficial aspects rather than completely suppress the inflammatory response. Studies suggest that MSCs engraftment at 7 days rather than immediately post-injury has strengthened their therapeutic efficacy. At present, most studies on the MSCs therapy focus on the acute and subacute phages post-SCI, while the therapeutic effect in the chronic phage still needs to be confirmed by further studies. Additionally, the immunoregulatory ability of MSCs may be enhanced after exposure to certain stimuli, so preconditioning of MSCs with certain cytokines before transplantation may lead to more effective immunomodulation.

## Glial Scar Following SCI

**Figure 3:**
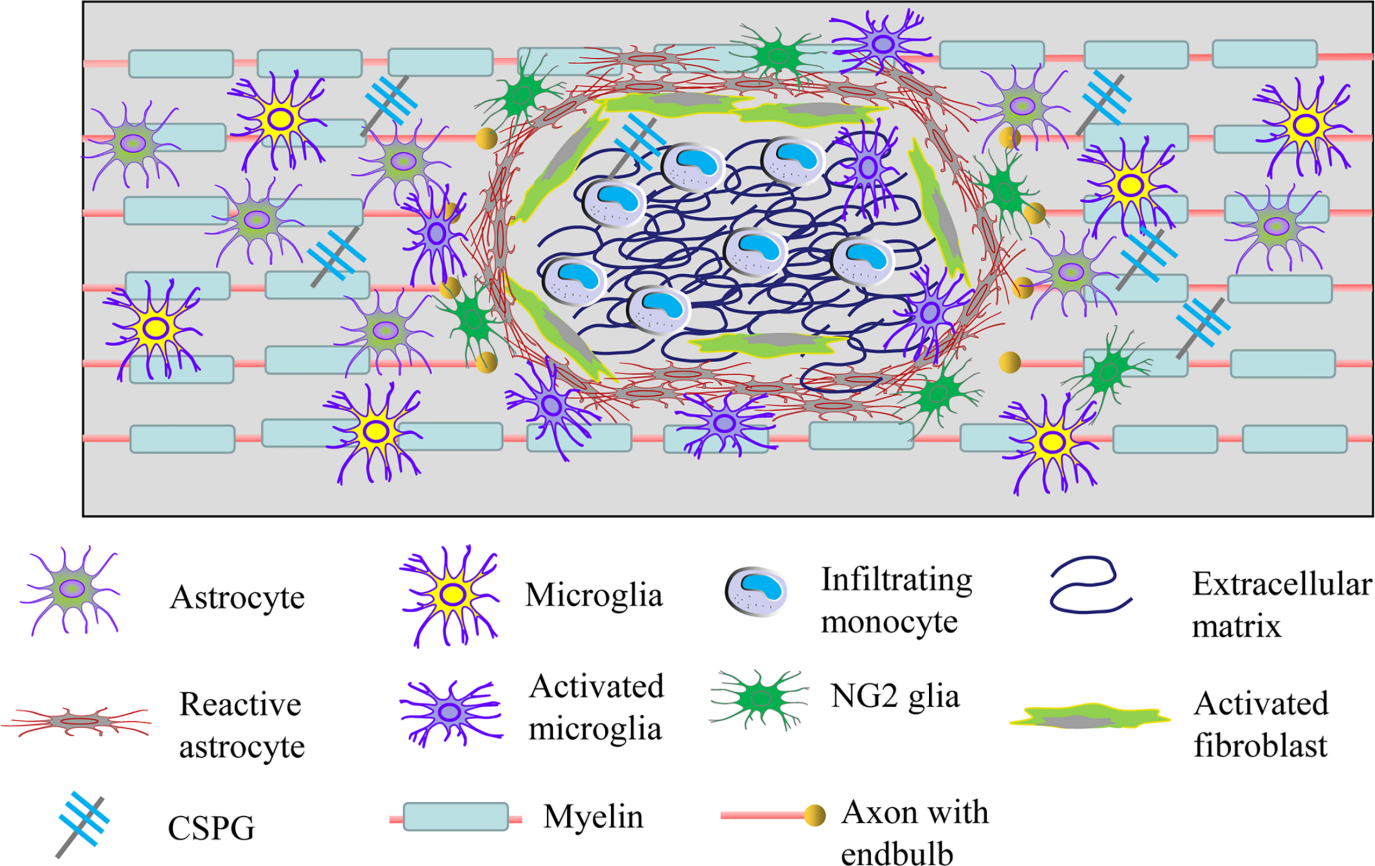
Cellular and extracellular components of glial scar after SCI. Resident astrocytes, microglia and NG2 glia become migratory, proliferate, activated and lead to the glial scar after injury. Meanwhile, fibroblasts and circulating immune cells infiltrate into the damaged tissue and then increase the deposition of extracellular matrix molecules including extracellular matrix and CSGP. Glial scar can isolate the damaged spinal cord tissue, but it also limits the axonal plasticity.

Once the spinal cord tissue gets injured, activated astrocytes, microglia and NG2 glia together form a dense border to isolate the severe damaged area (30). The lesion core contains a mixture of activated fibroblasts, monocyte macrophages, and extracellular matrix proteins (**Figure 3**) (106). Astrocytes are the most abundant glial cells in the CNS and play an important role in regulating blood flow, maintaining the integrity of the blood-brain barrier, modulating the plasticity and function of synapses, and keeping the balance of neuronal microenvironment (107, 108). After SCI, astrocytes will proliferate, hypertrophy and gradually migrate along the edge of the severely damaged tissue, interweaving around the lesion center to form the main component of glial scar (109–111). Hence, the main therapeutic strategies targeting glial scar focus on regulating the activation of astrocytes (112). Early on, glial scar was regarded as the major obstacle to neuronal axon regeneration in the CNS. Whereas, in recent years, growing evidence has shown that glial scar is also likely to promote axonal regeneration (113, 114). This difference is possibly explained by the fact that glial scar plays a protective role in the early stage of injury, but hinders the repair of the CNS in the later stage (115, 116).

### Adverse Effects of the Glial Scar

During the process of damaged nerve tissue healing, the adverse effect of reactive astrocytes on CNS functional recovery is mainly due to the glial scar that constitutes a physical barrier to axonal regeneration, making it difficult for axons to extend across the lesion area (117). In addition, chondroitin sulfate proteoglycans (CSPGs), the main chemical components of glial scar, including proteoglycans, versican, neurocan and brevican, mainly expressed by reactive astrocytes, can largely limit the axonal regrowth and sprouting as well as myelin sheath regeneration post-SCI (118–121). McKeon et al. (122) demonstrated that CSPGs might limit the growth of CNS neurites *in vitro*. Moreover, Takeuchi et al. (123) found that mice performed with a gene knockout for CS N-acetylgalactosaminyltransferase-1, a key enzyme of CSPGs synthesis, or treated with chondroitinase ABC, a kind of CSPGs degrading enzyme, had better motor function as well as axonal regeneration. Protein tyrosine phosphatase σ (PTPσ), a receptor of CSPGs, can inhibit the axonal regeneration while combined with CSPGs (124, 125). Whereas, blocking of this receptor by membrane-permeable peptide mimetic can not only attenuate this effect, but also promote functional recovery of the motor and urinary systems (124).

Considering the adverse effects of glial scar, the specific mechanisms of glial scar formation have been studied in depth. It was found that after SCI, locally increased TGF-β could induce the proliferation of astrocytes and the expression of CSPGs *via* activating the Smads signaling (116, 126, 127). However, these effects could be prohibited by TGF-β receptor blockers as well as taxol. The potential mechanism might be that taxol counteracted the translocation of Smad2/3 to the nucleus, thereby eliminating the effects of TGF-β signaling (128). Furthermore, studies have documented that inhibition of both JAK/STAT3 and JNK/c-Jun pathways can prevent the activation and proliferation of astrocytes, thereby reducing the formation of glial scar and facilitating the functional recovery (129).

### Beneficial Effects of the Glial Scar

The local lesions after SCI are mainly characterized by ion imbalance, free radical production, glutamate accumulation, excessive production of ROS and Reactive Species (RNS) (4). In addition, the activation of infiltrating peripheral immune cells and colonized microglia induces excessive inflammatory responses causing damage to more normal surrounding cells and tissues (130, 131). However, early glial scar can confine the inflammatory cells and various toxic molecules to the damaged tissue area, thereby protecting the healthy spinal cord tissue from inflammatory and toxic effects (4). Research showed that lack of reactive astrocytes after SCI could result in failure of BSCB repair, pronounced inflammatory response and demyelination, neuronal and oligodendrocyte degeneration, along with obvious motor defect, suggesting that the early loss of astrocytes is likely to be a main contributing factor to the secondary injury post-SCI (132). Furthermore, using a gene-targeted knockout approach in adult mice SCI models, Anderson et al. (133) showed that preventing astrocyte scar formation, reducing scar-forming astrocytes or deleting chronic astrocyte scar all did not result in spontaneous regrowth of sensory or serotonergic axons. In contrast, continuous local delivery of required axon-specific growth factors not present in SCI lesions through hydrogel depots under the condition of glial scar formation significantly induced axon regeneration, while preventing astrocyte scar formation significantly weakened this effect (133–135). However, as mentioned above, robust and persistent inflammation can lead to extensive cell destruction and severely inhibit axonal regeneration. The assumption that glial scars benefit axonal regeneration by only removing astrocytes may be oversimplified because it ignores that glial scars limit the horrific damage caused by the spread of inflammation. In addition, an increasing body of evidences has uncovered that the glial scar is also composed of other cellular and extracellular components beyond astrocytes, and that simply removing a specific cell type to affect the repair process may lead to inaccurate conclusions. The dual nature of glial scars in SCI has gradually attracted people’s attention, but a large number of studies are still needed to comprehensively and correctly understand their advantages and disadvantages.

## Role of MSCs on Glial Scar

Numerous experimental results have shown that MSCs transplantation into SCI animals has achieved striking therapeutic efficacy, along with bright future in clinical trials as well (136, 137) (**Tables 1**, **2**). These results may be closely related to the regulation of glial scar formation after SCI by MSCs (160). Okuda et al. (146) demonstrated that bone marrow-stromal cell sheets transplantation into SCI rats could not only inhibit the formation of glial scar but also provide a fantastic micro-environment for axonal regeneration and functional recovery by affecting the morphology of reactive astrocytes. In addition, chitosan (CS) based hydrogels loaded with MSCs transplantation into SCI mice effectively improved the survival rate of stem cells, which enabled them to release large amounts of growth factors and anti-inflammatory cytokines at the injured site to support nerve tissue repair, and significantly reduced glial scar, thereby creating a permissive environment for axonal growth and nerve regeneration (145). Similar situations can be seen not only in small animals, but also in large-animals SCI models. For example, transplanting neuroregen scaffold containing human umbilical cord-derived mesenchymal stem cells (UC-MSCs) into the lesion area of the canine chronic SCI models showed that they could significantly reduce the glial scar formation and promote neuronal regeneration, and ultimately improve locomotor recovery (161). As described above, TGF-β can mediate the glial scar formation by activating the Smads signaling in astrocytes (128). Studies have revealed that hepatocyte growth factor (HGF) overexpressing MSCs transplantation into rat SCI models could significantly reduce the levels of TGF-β, the activation of astrocytes, along with lower levels of CSPGs around hemisection lesions, which together promoted axonal growth and motor function recovery (162). In addition, LV et al. (144) found that BM-MSCs could also reduce activation of TGF-β/Smads signaling in astrocytes to promote recovery of motor function, which might be explained by the fact that BM-MSCs inhibited the formation of glial scar after injury and induced axonal regeneration. Notably, we found that after PB-MSCs transplantation into the SCI contusion rats, astrocytes bio-marker GFAP increased significantly 2 weeks after transplantation but decreased significantly 4 weeks later (103). Hence, we suspect that PB-MSCs transplantation can promote the glial scar formation in the early stage, which is beneficial for restricting the spread of various toxic molecules and immune cells. However, they could inhibit the formation of glial scar in the later stage, thus creating a permissive environment for axon regeneration. However, more studies are needed to confirm this hypothesis.

**Table 1:** Evaluation of MSCs therapy in SCI animals.

MSCs sources	Way	Model	Effect	Molecular mechanism	Refs.
Rat bone marrow-derived	Local injection	Rat	Promote functional recovery	Up-regulate the ratio of M2/M1 macrophages and the levels of associated cytokines	(76)
Rat bone marrow-derived	Local transplantation	Rat	Promote functional recovery	Reduce macrophage/microglia and T lymphocyte recruitment	(138)
Human cord blood-derived	Local transplantation	Mouse	Improve functional recovery	CCL2 secreted by MSCs induce the formation of M2 macrophages	(139)
Rat bone marrow-derived	Local transplantation	Rat	Promote functional recovery	Decrease macrophage/microglia infiltration and the expression levels of TNF-α and IL-1β at the damaged site	(140)
Human cord blood-derived	Local injection	Mouse	Alleviate neuropathic pain and promote functional recovery	Decrease macrophage/microglia activation, and the expression levels TNF-α and IL-6 at the damaged site	(141)
Human cord blood-derived	Local injection	Rabbit	Promote functional recovery	Anti-inflammatory, anti-astrocyte proliferation, anti-apoptosis and axonal preservation	(142)
Human cord blood -derived	Local injection	Mouse	Improve motor function, myelin, and nerve cell survival	Reduce the expression of IL-7, IFN-γ, and TNF-α but increase IL-4 and IL-13 expression, promote the activation of M2 macrophages	(143)
Rat bone marrow-derived	Local injection	Rat	Improve the spinal function	Reduce astrocyte proliferation and glial scar formation	(144)
Rat bone marrow-derived	Intravenous injection	Rat	Improve functional behavioral recovery	Promote angiogenesis, attenuate neuronal cells apoptosis, suppress the activation of A1 astrocytes and the formation of glial scar, attenuate lesion size, suppress inflammation, promote axonal regeneration	(86)
Rat peripheral blood-derived	Local injection	Rat	Promote functional recovery	Activate Tregs, inhibit Th17 cells, increase the expression levels of TGF-β and decrease the IL-6, IL-17a and IL-21 expression	(103)
Mouse bone marrow-derived	Local transplantation	Mouse	Promote functional recovery	Promote neuronal regeneration, limit the formation of glial scar, reduce cell death at the injured site	(145)
Rat bone marrow-derived	Local transplantation	Rat	Ameliorate the hindlimb locomotor function	Promote axonal regeneration, reduce glial scars formation	(146)

**Table 2:** MSCs with therapeutic potential for SCI patients.

Intervention	Transplantation	Dose(number)	Effect	Adverse reactions	Refs.
Autologous AD-MSCs	Intrathecal	9 ×10^7^	Improve motor, sensory, and sphincter control, no changes in areas of spinal damage	3 out of 14 patients have urinary tract infection, headache, nausea, and vomiting, no serious adverse events	(147)
Autologous BM-MSCs	Intramedullary	1.6×10^7^ to 3.2×10^7^	Limited efficacy	Safe, no adverse effects	(148)
Allogeneic UC-MSCs	intrathecal	10 x 10^6^	Improve pinprick sensation compared with placebo	Safe, no significant side effects	(149)
Autologous BM-MSCs	Intramedullary	300 x 10^6^	Improve urodynamics, anorectal pressure, neurophysiology, reduce spasms and neuropathic pain	Safe, no obvious adverse events	(150)
Autologous BM-MSCs	Intramedullary	5×10^6^	Improve tactile sensitivity, lower limbs motor function, AISA scores, and urologic function	Safe, low-intensity pain at the incision site	(151)
Allogeneic UC-MSCs	intrathecal	4 x 10^7^	Improve movement, self-care ability and muscular tension, increase maximum urine flow rate and maximum bladder capacity, reduce residue urine volume and maximum detrusor pressure	Safe, no obvious adverse reactions	(152)
Autologous AD-MSCs	Intravenous	4x10^8^	Improve the ASIA sensory scores, no significant differences in the pulmonary function test, SCIM, and visual analog scale.	Safe, no serious complications	(153)
Autologous BM-MSCs	Intrathecal	1x10^6^/kg	Various patterns of recovery, no significant changes in ASIA rating scale	Safe, no serious adverse events	(154)
Autologous BM-MSCs	Intrathecal	7x10^5^ to 1.2x10^6^	Improve neurological function	Safe, no any adverse reaction and complication	(155)
Autologous BM-MSCs	Intramedullary	200x10^5^	Improve movement, light touch, pin prick sensory, residual urine volume, and AISA scores	No sign of tumor, a few mild adverse reactions like headache and dizziness	(156)
Autologous BM-MSCs	Intrathecal	2×10^6^ to 7.71×10^6^/kg	N/A	Headache and nonspecific tingling sensation, no serious adverse event such as inflammation of spinal cord, cerebrospinal fluid infection, meningitis or tumor	(157)
Autologous BM-MSCs	Intrathecal	120×10^6^	Improve sensitivity, motor function, spasms, neuropathic pain, sexual function or bladder and bowel control	Headaches and pain in the area of the lumbar puncture, no other severe adverse events	(158)
Autologous BM-MSCs	Intrathecal	1.54×10^8^	Improve ASIA scores from A to C/D (from 112 to 231 points), expand the sensation level from Th1 to L3-4, restore the ability to control the trunk, bladder filling sensation, bladder control, and anal sensation	No neither early adverse events like infection, fever and pain, nor other late adverse events such as cancer	(159)

UC-MSCs, umbilical cord‐derived mesenchymal stem cells; BM-MSCs, bone marrow-derived mesenchymal stem cells; AD-MSCs, adipose-derived mesenchymal stem cells, ASIA, American Spinal Injury Association; SCIM, spinal cord independence measure.

## Clinical Application and Challenges for MSCs Therapies of SCI

Early encouraging basic researches on the therapeutic effects of MSCs have aroused considerable interest in examining their potential to facilitate functional recovery post-SCI. At present, there are 35 clinical trials of MSCs for SCI registered on clinicaltrials.gov. Although MSCs for SCI are still in the early stages of clinical transformation and few important literatures have been published so far, current results of the trials are promising. For example, Hur et al. (158) successfully isolated adipose-derived mesenchymal stem cells (AD-MSCs) from patient’s adipose tissue and intrathecally administered them to each of 14 patients by lumbar tapping. Over the 8 months of follow-up, five patients showed improvement in neurological recovery and two patients showed voluntary anal contraction improvement. According to the ASIA sensory scores, sensory improvement was observed in ten patients and sensory deterioration in one patient, and no septal changes in the size of the lesions and no signs of tumor or calcification were found in MRI after AD-MSCs transplantation. Moreover, none of the patients had any serious adverse events associated with intrathecal injection of AD-MSCs while urinary tract infection, headache, nausea, and vomiting were observed in three patients. The safety and efficacy of autologous BM-MSCs transplantation in patients with subacute SCI were evaluated in a nonrandomized phase I/II controlled clinical trial performed by Karamouzian et al. (154). In their study, 11 patients with T1-L1 complete SCI were enrolled. After 6 months, five patients out of 11 (45.5%) in the cell therapy group and three patients in the control group (15%) exhibited remarkable recovery (ASIA from A to C), and no significant adverse reaction and complications were observed in any patients. In another clinical trial, Albu et al. (148) conducted a phase 1/2a clinical trial of allogeneic MSCs transplantation in ten patients with chronic complete SCI (ASIA A) at dorsal level (T3-11). These patients were randomly designed to receive intrathecal injection of placebo or UC-MSCs and were then switched to the other arm at 6 months. During the follow-up of 12 months, non-serious side effects such as headache associated with spinal infusion were found in only two patients. The sensory perception in the dermatomes below the level of SCI was significantly promoted following MSCs transplantation compared with placebo, but without significant changes in spasticity, motor, bowel and bladder function. These existing clinical results preliminatively suggest that MSCs transplantation is safe and effective in the treatment of SCI, but the limitations of these studies should be acknowledged. Due to the small number and heterogeneity of patients, reliable analysis of the efficacy is not possible to make. Additionally, it is practically difficult to establish a control group because most patients who volunteer for such studies convey a strong desire to be included in the treatment group. Many aspects of MSCs therapy need to be clearly defined if they are to be fully translated into the clinic. For example, MSCs from different sources may differ in their reparative potential for neural tissue, meaning that a certain type of MSCs may be more suitable for treating SCI than others. The ways of administration of engrafted MSCs also have a critical impact on their distribution in specific tissues, and their therapeutic efficacy in damaged areas. At present, the most generally used administration methods for SCI mainly include local and intravenous transplantation, but which transplantation approach can achieve the maximum benefit still needs to be further clarified.

Although MSCs have shown great therapeutic potential in both animal and human trials of SCI, there are still some challenges that require to be figured out before MSCs can be extensively applied in clinic. MSCs may play a therapeutic role through immunosuppressive mechanisms, but this is also a double-edged sword, as they can enhance tumor growth by suppressing the immune response (163). Multiple studies have revealed that MSCs can also actively participate in tumor progression and migration in various cancer types by activating diverse pathways (164). In addition, MSCs can also be guided towards the tumor site, which not only allows MSCs to promote tumor growth and angiogenesis by secreting various pro-angiogenic cytokines such as vascular endothelial growth factor (VEGF), but also causes the malignant transformation of MSCs (163). It is worth noting that gene modification may be an effective method to enhance the immunoregulatory capacity and survival ability of MSCs, but the insertion of specific genes will destroy the genome of MSCs and then may induce tumorigenesis in recipients. Hence, its long-term tumorigenicity remains to be further observed. Studies have shown that telomere length of MSCs can be shortened in the long-term culture process, resulting in a gradual decline in cell proliferation and a significant increase in the chance of malignant transformation (165). Hence, it may be very important to use the non-aging MSCs for clinical treatment. Crucially, the quality and safety of MSCs from different laboratories vary greatly, so there is an urgent need to develop general guidelines for the preparation and storage of MSCs.

## Conclusion

SCI can lead to multifaceted cellular and molecular reactions, and these various changes are the pathogenesis basis of secondary injury. Among all of the changes, the inflammation and glial scar formation are the major barriers to neuronal anatomical and functional repair, directly determining the disease progression and prognosis, thus forcing researchers to explore effective treatment measures for these responses. In recent years, numerous preclinical and clinical studies have demonstrated the efficacy and safety of MSCs in the treatment of SCI, but its long-term tumorigenicity remains to be further observed. Here, we mainly conclude that MSCs may offer therapeutic potentials for damaged spinal cords by regulating neuroinflammation and glial scar formation. However, more studies are needed to explore the specific mechanisms by which MSCs inhibit inflammation and glial scar formation post-SCI.

## Author Contributions

Q-MP drafted the manuscript. TZ and J-CP were responsible for the design and conception of the study. All authors discussed and edited the manuscript, and read and approved the final version.

## Funding

This work was supported by grants from the National Natural Science Foundation of China (No.81960299), and the Guizhou Province Biotherapy Talent Base Construction Project ([2013] No.5).

## Conflict of Interest

The authors declare that the research was conducted in the absence of any commercial or financial relationships that could be construed as a potential conflict of interest.

## Publisher’s Note

All claims expressed in this article are solely those of the authors and do not necessarily represent those of their affiliated organizations, or those of the publisher, the editors and the reviewers. Any product that may be evaluated in this article, or claim that may be made by its manufacturer, is not guaranteed or endorsed by the publisher.
